# Plazomicin Is Active Against Metallo-β-Lactamase-Producing Enterobacteriaceae

**DOI:** 10.1093/ofid/ofz123

**Published:** 2019-03-12

**Authors:** Alisa W Serio, Tiffany Keepers, Kevin M Krause

**Affiliations:** Department of Clinical Microbiology, Achaogen, Inc., South San Francisco, California

**Keywords:** aminoglycoside, metallo-β-lactamase, plazomicin

## Abstract

Plazomicin is an aminoglycoside that was approved in June 2018 by the US Food and Drug Administration for the treatment of complicated urinary tract infections, including pyelonephritis, due to *Escherichia coli*, *Klebsiella pneumoniae*, *Enterobacter cloacae*, and *Proteus mirabilis*. Plazomicin was engineered to overcome the most common aminoglycoside resistance mechanism, inactivation by aminoglycoside-modifying enzymes, but is not active against the less common 16S ribosomal RNA methyltransferases (16S-RMTase), which confer target site modification. As an aminoglycoside, plazomicin maintains activity against Enterobacteriaceae that express resistance mechanisms to other antibiotic classes, including metallo-β-lactamases. Therefore, in the absence of a 16S-RMTase, plazomicin is active against metallo-β-lactamase-producing Enterobacteriaceae.

Plazomicin is an aminoglycoside developed from a sisomicin scaffold via chemical modification to evade the most common aminoglycoside resistance mechanisms in Enterobacteriaceae, aminoglycoside-modifying enzymes (AMEs) [1, 2]. The only AMEs known to impact plazomicin activity are AAC(2)-Ia, which is only found on the chromosome of *Providencia stuartii*, and AAC(2′′)-IVa, which is only found in *Enterococcus* spp. [1]. Plazomicin was approved by the US Food and Drug Administration (FDA) in June 2018 for the treatment of complicated urinary tract infections, including pyelonephritis, due to *Escherichia coli*, *Klebsiella pneumoniae*, *Enterobacter cloacae*, and *Proteus mirabilis* [3].

Plazomicin is potent against multidrug-resistant (MDR) Enterobacteriaceae, because, as an aminoglycoside, its activity is not impacted by resistance mechanisms to other antibiotic classes such as β-lactamases and carbapenemases, including metallo-β-lactamases (MBLs), as well as fluoroquinolone and colistin resistance mechanisms [4]. However, plazomicin lacks activity against organisms that encode 16S ribosomal RNA methyltransferases (16S-RMTases); to date, this is the only resistance mechanism reported to result in plazomicin minimum inhibitory concentrations (MICs) ≥64 μg/mL for Enterobacteriaceae spp. [1, 4, 5]. Thus, in the event that a 16S-RMTase is found on a plasmid with other resistance genes, such as an MBL, plazomicin will be inactive. As a result of this, some reports suggest that plazomicin has no activity against MBL producers.

Here we present data on the activity of plazomicin against MBL-producing carbapenem-resistant Enterobacteriaceae (CRE) clinical isolates. The data were compiled from 9 different studies, including 4 years (2014–2017) of global surveillance (Antimicrobial Longitudinal Evaluation and Resistance Trends [ALERT] global surveillance program conducted by JMI, North Liberty, IA; data on file), and 5 regional studies, with isolates collected from 19 different countries [5–9]. The regional studies were designed to assess the activity of plazomicin against CRE collected between 2006 and 2009 from the United Kingdom [5], against MDR *E. coli*, *K. pneumoniae,* and *E. cloacae* collected between 2008 and 2010 from Athens, Greece [6], against carbapenem-resistant *K. pneumoniae* collected between 2014 and 2016 from a nationwide collection in Greece [7], against CRE collected between 2010 and 2013 from the United States [8], and against carbapenemase-producing Enterobacteriaceae collected between 2013 and 2015 from Brazil [9]. These studies combined with the ALERT surveillance study provide a collection of data for isolates primarily from Europe (63.1%), followed by Latin America (26.6%), Asia Pacific (8.4%), and North America (3.5%). The majority of isolates were *K. pneumoniae* (63.3%), followed by unspeciated CRE (24.4%), *E. cloacae* (5.9%), and *E. coli* (2.5%). The other 4.9% of isolates were *Citrobacter freundii, Enterobacter aerogenes, Klebsiella oxytoca, Morganella morganii, Providencia rettgeri,* and *Providencia stuartii*. The data selected for this compilation were unbiased and, to our knowledge, represent the only complete data sets available that specifically report the plazomicin MIC distribution for an entire collection of CRE molecularly characterized for carbapenemases, including MBLs.

Figure 1 shows the plazomicin MIC distribution (range of MICs, ≤0.12 to >64 μg/mL; MIC_50_ and MIC_90_, 1 and >64 μg/mL, respectively) against the collection of MBL producers (n = 488); 76.4% (373/488) of isolates were susceptible to plazomicin at the FDA breakpoint of ≤2 μg/mL. Among the 115 isolates not susceptible to plazomicin, 98 were molecularly characterized, and the majority (86/98, 87.8%) of the isolates were found to have a 16S-RMTase. The plazomicin resistance mechanisms in the remaining isolates (n = 19), which included 13 with intermediate MICs (4 μg/mL) and 6 with resistant MICs (8–16 μg/mL), are unknown. These isolates were primarily *K. pneumoniae* and were from Greece (n = 9), Brazil (n = 7), the United States (n = 1), and Mexico (n = 2). Only 19.7% (96 of 488) of isolates had a plazomicin MIC ≥64 μg/mL.

**Figure 1.**
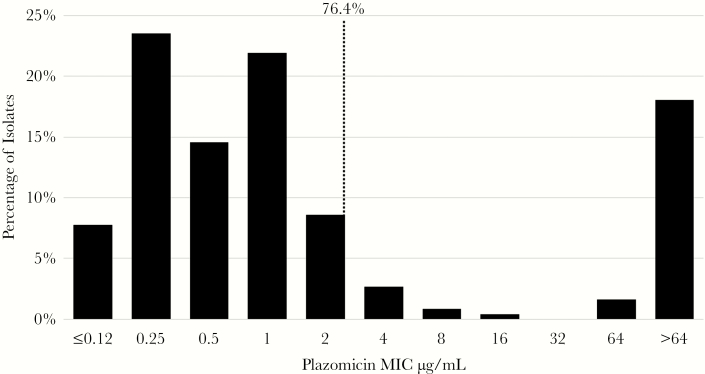
Minimum inhibitory concentration distribution of plazomicin against 488 metallo-β-lactamase-producing carbapenem-resistant Enterobacteriaceae global clinical isolates. Dotted line indicates plazomicin-susceptible breakpoint of ≤2 µg/mL [3]. Isolates originated from 19 countries: Greece (n = 215), Brazil (n = 89), United Kingdom (n = 36), Mexico (n = 39), Turkey (n = 22), United States (n = 11), Thailand (n = 17), Malaysia (n = 11), Belarus (n = 8), Poland (n = 9), Philippines (n = 7), Russia (n = 6), Australia (n = 4), Germany (n = 5), Italy (n = 3), Taiwan (n = 2), Ukraine (n = 2), and Slovenia (n = 1). Data sources were as follows: 2014–2017 ALERT JMI global surveillance data on file: NDM = 132, VIM = 22, IMP = 8 [5]; NDM = 17, IMP = 13, VIM = 5 [6]; VIM = 119 [7]; NDM = 52, VIM = 35 [8]; NDM = 1 [9]; NDM = 80, IMP = 3, VIM = 1. Abbreviation: MIC, minimum inhibitory concentration.

Approximately half (57.8%) of MBL producers were New Delhi metallo-β-lactamase (NDM)-positive, and 66% of these isolates were susceptible to plazomicin. It has been shown that NDM is frequently co-expressed with 16S-RMTases, and plazomicin, like all aminoglycosides, is not active against isolates that express a 16S-RMTase. Among the 488 isolates in this data set, 282 isolates had an NDM gene, and of these isolates, 64 had plazomicin MICs ≥64 μg/mL, indicative of 16S-RMTase production. These 64 isolates represent 22.7% of the NDM-positive isolates and 13.1% of the total isolates. In addition, 37.3% of isolates were Verona integron-encoded metallo-β-lactamase (VIM)-positive, and 89.6% of these isolates were susceptible to plazomicin. Further, a small percentage (4.9%) of isolates were Imipenemase metallo-β-lactamase (IMP)-positive, and 100% of these isolates were susceptible to plazomicin.

As described above, *K. pneumoniae* was the predominant species in this collection and was also the predominant NDM-positive species (62%, 175/282) and VIM-positive species (72%, 131/182). Nonspeciated CRE accounted for the majority of the remaining MBL-positive organisms (IMP: 66.7%, 16/24; NDM: 34.4%, 97/282; VIM: 3.3%, 6/182). Regionally, Brazil (30% 85/282), Greece (20.2% 57/282), and Mexico (12.8%, 36/282) had the most NDM-positive organisms, whereas Greece also had the predominant number of VIM-positive organisms (86.8%, 158/182).

A total of 196 isolates were also assessed for susceptibility to other aminoglycosides; this was determined in the surveillance program (data on file) and 1 independent study [5]. Using CLSI breakpoints, 46.9% (92/196) of isolates were susceptible to amikacin (MIC ≤ 16 µg/mL), 32.1% (63/196) were susceptible to gentamicin (MIC ≤ 4 µg/mL), and 6.1% (12/196) were susceptible to tobramycin (MIC ≤ 4 µg/mL). Using EUCAST breakpoints, 29.6% (58/196) of isolates were susceptible to amikacin (MIC ≤ 8 µg/mL), 30.6% (60/196) were susceptible to gentamicin (MIC ≤ 2 µg/mL), and 6.1% (12/196) were susceptible to tobramycin (MIC ≤ 4 µg/mL). Comparatively, 57.7% (113/196) of these isolates were susceptible to plazomicin using FDA breakpoints (MIC ≤ 2 µg/mL).

In conclusion, plazomicin was active against >75% of all MBL producers tested. The authors believe that the combined studies presented here represent a large data set; however, additional studies could provide further insight into the activity of plazomicin against MBL producers and highlight that plazomicin is indeed active against most MBL producers in the absence of 16S-RMTase co-expression. Based on these data, plazomicin is expected to retain activity against the vast majority of MBL-producing Enterobacteriaceae when applying the breakpoint (2 μg/mL) from the US Prescribing Information [3]. Therefore, in the clinical setting, the susceptibility of plazomicin to MBL-producing Enterobacteriaceae should be based on antimicrobial susceptibility test results rather than a presupposition that plazomicin is inactive against these isolates. At this time, there is no known diagnostic available that can differentiate plazomicin-susceptible MBL-producing isolates from plazomicin-resistant MBL-producing isolates in the absence of a susceptibility result.
